# Zika Virus Persistence and Higher Viral Loads in Cutaneous Capillaries Than in Venous Blood

**DOI:** 10.3201/eid2311.170337

**Published:** 2017-11

**Authors:** Séverine Matheus, Franck de Laval, David Moua, Christophe N’Guyen, Enguerrane Martinez, Dominique Rousset, Sébastien Briolant

**Affiliations:** Institut Pasteur de la Guyane, Cayenne, French Guiana (S. Matheus, D. Moua, D. Rousset);; Military Centre for Epidemiology and Public Health, Marseille, France (F. de Laval);; French Armed Forces Health Service, Cayenne (F. de Laval, E. Martinez);; French Armed Forces Biomedical Research Institute, Marseille (C. N’Guyen, S. Briolant)

**Keywords:** Zika virus, Zika, venous samples, capillary blood samples, persistence, vector-borne infections, viruses, French Guiana

## Abstract

We collected venous and capillary serum samples from 21 Zika virus‒infected patients on multiple days after symptom onset and found RNA load was higher and median duration of virus detection significantly longer in capillary than in venous blood. These findings raise questions about the role of the capillary compartment in virus transmission dynamics.

Zika virus, belonging to the family *Flaviviridae* and genus *Flavivirus*, is transmitted to humans by mosquito bites but can also be contracted through sexual and vertical transmission (*1*). Zika virus was first detected in Brazil in 2015 and has since spread with the same speed as chikungunya virus through South and Central America and the Caribbean Islands, despite a shorter and lower viremia in humans and a longer replication period in the vector (*2**,**3*). As with other arboviruses, Zika virus viremia is commonly measured in venous blood, even though mosquitoes introduce virus into cutaneous capillary blood. We conducted a prospective descriptive study with Zika virus patients during the Zika virus epidemic in French Guiana to evaluate the kinetics of Zika virus RNA load in serum samples collected sequentially from venous and skin capillary blood.

The study population comprised 21 symptomatic and consenting Zika virus patients infected during March‒September 2016. We confirmed Zika virus infection by real-time reverse transcription PCR (RT-PCR) of serum and urine samples provided by patients during the first few days after symptom onset. We obtained serum samples from the venous and cutaneous capillary blood (collected from the fingertip) sequentially 1‒18 days after the onset of symptoms. The median age of the population was 40 (range 28–63) years and the sex ratio (male:female) was 1.6. We observed no co-morbidities, and all participants were found to be free of dengue and chikungunya virus infections by methods previously described (*4**,**5*).

We extracted RNA from 150-µL samples using the QIAamp Viral RNA kit (QIAGEN, Hilden, Germany) and performed Zika virus RNA amplification using the RealStar Zika Virus RT-PCR Kit 1.0 (Altona Diagnostics GmbH, Hamburg, Germany) according to the manufacturer’s instructions. We performed Zika virus RNA quantification using a reference strain provided by the European Zika Virus Archive (SKU no. 001N-01648) and estimated the Zika virus RNA load as log_10_ copies per milliliter Technical Appendix Table).

Zika virus RNA loads in capillary blood correlated with those in venous blood (Spearman correlation test, *r* = 0.54, p<0.0001) but were significantly higher in the capillary samples (Wilcoxon signed rank test, p = 0.0003), except for 3 patients (nos. 2, 13, and 21; Technical Appendix Table). The median duration of Zika virus detection after symptom onset was significantly greater in capillary blood than in venous blood (p = 0.005 by log rank test; hazard ratio = 2.99, 95% CI 1.39–6.43), even though the duration of detection in capillary blood was underestimated; RNA was still detectable in the last capillary blood samples taken from 8 patients (nos. 1, 5, 6, 8, 11, 15, 16, and 19; Technical Appendix Table). The duration of Zika virus RNA detection was greater in capillary than in venous blood for 12 (57%, 95% CI 34%–78%) of the 21 patients (nos. 1, 3, 5, 6, 7, 8, 11, 14, 15, 16, 19, and 20). The maximum duration of RNA detection in capillary blood samples was 18 days after the onset of symptoms (seen with patient no. 19) with a load of 1.9 log_10_ copies/mL. The duration of detection was equal between the 2 compartments for 7 (33%, 95% CI 15%–57%) of 21 patients (nos. 4, 9, 10, 12, 13, 17, and 18) and longer in venous blood for 2 (10%, 95% CI 1%–30%) of 21 patients (nos. 2 and 21) (Technical Appendix Table).

These data raise questions about the consequences of longer persistence and higher loads of Zika virus RNA in the cutaneous capillary blood compartment, although we did not test Zika virus replication capacity. The higher load and longer detection of Zika virus RNA in this compartment might be attributable to Zika virus replication in permissive cells of the skin (e.g., human dermal fibroblasts and epidermal keratinocytes); capillaries; or both (*6*). If the Zika virus RNA observed in the serum samples taken from the capillary compartment reflects the presence of infectious virus particles, symptomatic Zika virus‒infected patients would need to be shielded from mosquitoes for a longer period than is currently practiced to limit potential vectorborne transmission.

This study comparing the kinetics of Zika virus RNA load between the venous and capillary compartments highlights the need to further investigate the infectivity and pathophysiology of the virus located in the often neglected capillary compartment. These findings provide new information on this biologic compartment, which plays a key role in vectorborne transmission and transmission dynamics. Moreover, these observations, if validated with more patients and extended to other vectorborne infections, will be vital for preventing and controlling the transmission of Zika virus and other arboviruses.

Technical Appendix Kinetics of Zika virus load in venous and capillary blood samples from 21 patients, French Guiana, 2016.

## References

[1] Petersen LR, Jamieson DJ, Honein MA. Zika virus. N Engl J Med. 2016;375:294–5. 27355409 10.1056/NEJMc1606769

[2] Waggoner JJ, Gresh L, Vargas MJ, Ballesteros G, Tellez Y, Soda KJ, et al. Viremia and clinical presentation in Nicaraguan patients infected with Zika virus, chikungunya virus, and dengue virus. Clin Infect Dis. 2016;63:1584–90. 10.1093/cid/ciw589 27578819 PMC5146717

[3] Chouin-Carneiro T, Vega-Rua A, Vazeille M, Yebakima A, Girod R, Goindin D, et al. Differential susceptibilities of *Aedes aegypti* and *Aedes albopictus* from the Americas to Zika virus. PLoS Negl Trop Dis. 2016;10:e0004543. 10.1371/journal.pntd.0004543 26938868 PMC4777396

[4] Callahan JD, Wu SJ, Dion-Schultz A, Mangold BE, Peruski LF, Watts DM, et al. Development and evaluation of serotype- and group-specific fluorogenic reverse transcriptase PCR (TaqMan) assays for dengue virus. J Clin Microbiol. 2001;39:4119–24. 10.1128/JCM.39.11.4119-4124.2001 11682539 PMC88496

[5] Panning M, Grywna K, van Esbroeck M, Emmerich P, Drosten C. Chikungunya fever in travelers returning to Europe from the Indian Ocean region, 2006. Emerg Infect Dis. 2008;14:416–22. 10.3201/eid1403.070906 18325256 PMC2570846

[6] Hamel R, Dejarnac O, Wichit S, Ekchariyawat P, Neyret A, Luplertlop N, et al. Biology of Zika virus infection in human skin cells. J Virol. 2015;89:8880–96. 10.1128/JVI.00354-15 26085147 PMC4524089

