# Supplementary material for: Zika Virus Persistence and Higher Viral Loads in Cutaneous Capillaries Than in Venous Blood
Source: Emerg Infect Dis. 2017 Nov;23(11):1910–1. doi: 10.3201/eid2311.170337 (PMC5652410; doi:10.3201/eid2311.170337)
Supplement: Technical Appendix — Kinetics of Zika virus load in venous and capillary blood samples from 21 patients, French Guiana, 2016. [file 17-0337-Techapp-s1.pdf]

Article DOI: https://doi.org/10.3201/eid2311.170337

Zika Virus Persistence and Higher Viral Loads in Cutaneous Capillaries Than in Venous Blood

Technical Appendix

Technical Appendix Table. Kinetics of Zika virus load in venous and capillary blood samples from 21 patients, French Guiana, 2016*

Patient no.	Age, y	Sex	Day after symptom onset	Cycle threshold		Zika virus load, log_{10} copies/mL	
				Venous blood	Urine	Venous blood	Capillary blood
1	40	M	3	33.26	32.77	3.8	NA
			8			<1.8	2.8†
2	45	M	3	33.26	32.77	2.8	<1.8†
			6			2.3	<1.8†
			7			<1.8	<1.8
3	59	F	2	36.75	34.02	2.6	3.9†
			3			<1.8	3.1†
			5			<1.8	<1.8
			10			<1.8	<1.8
4	34	M	2	38.01	28.41	2.2	3.7†
			4			<1.8	<1.8
			11			<1.8	<1.8
5	37	M	1	30.27	34.08	4.3	4.2
			3			<1.8	2.4
			7			<1.8	2.6
6	28	F	4	31.00	NA	4.6	NA
			5			4.1	4.2
			7			<1.8	2.8
7	42	M	2	34.29	36.88	3.6	4.8†
			3			<1.8	3.5
			5			<1.8	2.7
			8			<1.8	<1.8†
8	41	F	3	Negative	29.00	<1.8	3.7
9	34	F	2	36.92	35.00	2.9	3.1
			7			<1.8	<1.8†
10	41	M	3	35.35	33.63	3.3	3.4†
			5			<1.8	<1.8
			10			<1.8	<1.8
11	35	M	2	36.64	30.17	2.6	2.9
			3			<1.8	2.6
			5			<1.8	2.5
			8			<1.8	2.4
12	39	M	3	34.16	32.9	2.7	4.0†
			6			<1.8	<1.8†
			10			<1.8	<1.8†
13	39	M	3	36.97	27.63	2.5	2.4
			5			<1.8	<1.8
			10			<1.8	<1.8†
14	45	M	2	33.48	36.45	3.8	NA
			3			<1.8	3.3†
			6			<1.8	<1.8†
			8			<1.8	<1.8†
15	46	M	1	33.96	29.27	3.9	3.9†
			3			3.1	3.5
			5			NA	3.8†
			7			2.3	4.2
16	63	F	3	31.37	34.44	3.9	3.4
			8			<1.8	<1.8
			10			<1.8	2.5

Patient no.	Age, y	Sex	Day after symptom onset	Cycle threshold		Zika virus load, log_{10} copies/mL	
				Venous blood	Urine	Venous blood	Capillary blood
17	44	M	2	37.38	34.17	2.5	5.7†
			3			<1.8	<1.8†
			5			<1.8	<1.8†
			7			<1.8	<1.8†
18	32	F	1	30.92	34.70	4.3	NA
			3			3.0	3.1†
			7			<1.8	<1.8†
			15			<1.8	<1.8
19	35	M	4	Negative	36.56	<1.8	3.8†
			6			<1.8	3.2†
			8			<1.8	3.7
			18			NA	1.9
20	41	F	2	31.99	37.00	4.1	4.4†
			4			<1.8	3.7†
			7			<1.8	3.6
			14			<1.8	<1.8
21	35	F	1	34.91	NA	3.4	<1.8†
			3			<1.8	<1.8†
			6			<1.8	<1.8
			13			<1.8	<1.8†

*NA, not available; RT-PCR, reverse transcription PCR. Quantification was performed by real-time RT-PCR (RealStar Zika Virus RT-PCR Kit 1·0 CE; Altona Diagnostics GmbH, Hamburg, Germany) with a detection threshold of 1.8 log_{10} copies/mL.
†Volume of sample used for extraction was <50 µL.
